# Enhancing Radiation Safety Culture: Investigating the Mediating Role of Awareness Among Orthopedic Doctors and Operation Theatre Assistants

**DOI:** 10.7759/cureus.41704

**Published:** 2023-07-11

**Authors:** Junaid Khan, Bilal Khalid, Muhammad Zulfiqar Abbasi, Raja Adnan Ashraf, Kamran Asghar, Muhammad Nadeem Kashmiri, Kashif Tousif, Faizan Shahzad, Jawad Basit, Tehseen Haider, Haroon Shabbir, Abdul Rauf Khalid, Sajeel Saeed

**Affiliations:** 1 Orthopaedic Surgery, Benazir Bhutto Hospital, Rawalpindi, PAK; 2 Orthopaedic Surgery, Railway General Hospital, Rawalpindi, PAK; 3 Orthopaedic Surgery, Fauji Foundation Hospital, Rawalpindi, PAK; 4 Orthopaedic Surgery, Watim Medical College, Islamabad, PAK; 5 Medicine, Rawalpindi Medical University, Islamabad, PAK; 6 Medicine, Rawalpindi Medical University, Rawalpindi, PAK; 7 Cardiology, Rawalpindi Medical University, Rawalpindi, PAK; 8 Surgery, Rawalpindi Medical University, Rawalpindi, PAK

**Keywords:** risk exposure, fluoroscopy intervention, radiation, awareness, orthopedics

## Abstract

Introduction: The increasing use of minimally invasive orthopedic procedures has led to a greater reliance on fluoroscopy, resulting in elevated radiation exposure for surgeons. This study aimed to evaluate the knowledge, awareness, and daily practices of orthopedic surgeons regarding radiation safety in an academic hospital. Understanding radiation safety is crucial to minimize patient exposure and prevent adverse effects on surgeons.

Methods: This cross-sectional study was conducted at the Department of Orthopedics of different tertiary care hospitals in Rawalpindi, Pakistan. Data were collected prospectively for two years, and a total of 505 participants, including residents, consultants, and operation theatre assistants, completed a questionnaire. The questionnaire was validated by experts and covered information on fluoroscopy usage, frequency of surgeries, awareness of radiation safety, and protective measures. Ethical approval was obtained, and data were analyzed using SPSS version 26.0.

Results: The majority of participants were male (74.1%), and the sample included various ranks of orthopedic surgeons. Only 56.2% of participants were aware of the usage of fluoroscopy, and 40.2% had read some research on the topic. While 44.6% used lead aprons for radiation protection, the usage of other protective measures and dosimeters was limited. The mediation analysis showed an insignificant indirect association between the rank of orthopedic surgeons, number of surgeries performed, and fluoroscopy usage as a mediator. Awareness and reading research on fluoroscopy were significantly associated with radiation protection.

Conclusion: The knowledge, awareness, and daily practices of orthopedic surgeons regarding radiation safety in fluoroscopy use need improvement. The findings emphasize the importance of implementing training programs, providing radiation protection devices, and ensuring compliance with safety guidelines.

## Introduction

There is a growing focus on minimally invasive methods to reduce postoperative problems and injuries, as the number of older people needing orthopedic therapy increases. However, these procedures have led to a higher dependence on fluoroscopy by surgeons, which has increased their exposure to direct and dispersed ionizing radiation. In several orthopedic treatments, including trauma, reconstructive, and pediatric surgery, indirect anatomical observation is crucial. Radiation exposure is well-known to be detrimental [1,2].

Due to the established biological consequences of ionizing radiation, the use of fluoroscopy in the operating room raises hazards for orthopedic surgeons [2,3]. Radiation may have both deterministic (non-random) and stochastic (random) effects, depending on the dosage. While the risk of predictable consequences like cataracts, alopecia, migraines, skin ulceration, and infertility is minimal below a specific threshold, any dosage of radiation has the potential to cause cancer in radiosensitive tissues including the breast, lungs, thyroid, and red bone marrow [4]. According to the linear no-threshold model, any exposure to ionizing radiation is deemed equally dangerous, since the risk of cancer formation rises with increasing doses [3]. To reduce patient exposure during surgical procedures, orthopedic surgeons must have complete awareness of radiation safety.

Interestingly, orthopedic surgeons are less sensitive to radiation exposure to the hand than radiologists and cardiologists [5]. However, investigations done in England and Wales by Khan et al. revealed that basic surgical trainees had minimal understanding of the use of ionizing radiation in orthopedic trauma surgery, and even fewer were aware of the relevant literature [6].

The threat presented by ionizing radiation is exacerbated by the fact that it is unseen and intangible. Those orthopedic surgeons who must operate in the X-ray beam's direction are unfortunately exposed to radiation [7,8]. Many orthopedic surgeons do not get the same amount of radiation safety training as other medical professionals, despite these concerns. Previous researches by Tunçer et al., Saroki et al., and Nugent et al. demonstrated that orthopedic surgeons often lack enough understanding of the use and hazards of ionizing radiation, as well as the essential radioprotective measures to prevent radiation-related injury [7,9,10].

The aim of this research was to assess the knowledge, awareness, and everyday practices of orthopedic surgeons in an academic hospital regarding radiation safety. The research aims to address the research gap that exists in the understanding and implementation of radiation safety measures among orthopedic surgeons. Despite the increasing reliance on fluoroscopy in orthopedic procedures, which exposes surgeons to ionizing radiation, there is limited awareness around and training on radiation safety within the orthopedic field. Previous studies have highlighted the lack of understanding and knowledge among orthopedic surgeons regarding the use and hazards of ionizing radiation, as well as the necessary radioprotective measures. Therefore, this research aims to contribute to the existing body of knowledge by evaluating the current level of awareness and practices related to radiation safety among orthopedic surgeons, ultimately providing valuable insights to improve radiation safety protocols in orthopedic surgery.

## Materials and methods

Study design

This analytical, cross-sectional study was conducted at the Department of Orthopedics of different tertiary care hospitals in Rawalpindi, Pakistan. Data were collected prospectively for a duration of two years, i.e., from September 5, 2020, to September 4, 2022. A total of 505 participants were included in the study. The questionnaire was distributed among the residents, consultants, and operation theatre assistants (OTAs) working in orthopedic operation theatres of tertiary care hospitals. All the participants voluntarily filled out the questionnaire after informed consent.

Content validity

The questionnaire was sent to three experts from different domains of the university to check for content validity of the questionnaire. These experts were from the community medicine department and orthopedics department. These experts were then asked to assess the suitability of the questionnaire in terms of the expression of its instruction, test items, and whether its subtests were in accord with their respective fields. The experts then checked the quality of the questionnaire, and accordingly, it was decided that each item should be retained in the questionnaire.

Questionnaire

Information was recorded on printed questionnaires after taking informed consent from participants. The questionnaire consisted of all the information regarding the use and knowledge of the fluoroscope, the average number of days a surgeon does surgeries, etc. (Table 1).

**Table 1:** Radiation safety and fluoroscopy practices questionnaire

Questionnaire		
S. no.	Questions	Answers
1.	No. of days when you performed/assisted with surgery per week	1
		2
		3-5
		>5
2.	No. of surgeries performed/assisted with per week	5
		6-10
		11-15
		>15
3.	How many of your operations need fluoroscopy?	All operations
		>50%
		25%-50%
		<25%
4.	Average no. of fluoroscope images taken on an operative day	50
		50-100
		100-200
		>200
5.	Are you aware of the use of a fluoroscope?	Yes
		No
6.	Have you read any research on fluoroscopy?	Yes
		No
7.	Who operates the fluoroscope during the surgical procedure?	Technician
		Nurse
		Assisting surgeon
		None of the above
8.	How do you protect yourself from radiation during fluoroscopy?	Lead apron
		Personal protective equipment
		More than one of these
		None of above
9.	Have you ever had any of the following complaints?	Headache and fatigue
		Nausea/vomiting
		More than one of these
		None of these
10.	Do you use a dosimeter?	Yes
		No

This questionnaire was originally written in English, but the research investigators translated it into Urdu to make it easier to understand. This questionnaire was then back-translated to ensure its validity. To guarantee compliance and the clarity of the questionnaire, a pilot research with 30 participants was also carried out before the survey. These responses were not included in the final data.

Ethical approval

Before the start of the study, the Institutional Review Board (IRB) of Benazir Bhutto Hospital in Rawalpindi, Pakistan, granted ethical permission (RSRS-2020-S-022) for the research. The medical superintendents (MS) of Benazir Bhutto Hospital was formally requested for authorization to gather data. Consent was obtained before data collection, and participant anonymity was maintained throughout the investigation. The technique of data collection adhered to national and institutional ethical requirements, as well as the most current edition of the Helsinki Declaration. Each participant provided their informed consent. The anonymity and confidentiality of the data were maintained. The study adhered to the Strengthening the Reporting of Cohort Studies in Surgery (STROCSS) guidelines for cross-sectional research reporting.

Sample size

Using the WHO sample size calculator, a sample size of 505 individuals was determined; with a population size of 735 and a 95% confidence level, the expected proportion (P) of the population with adherence to standard operating procedures (SOPs) was taken as 0.5, and the margin of error (d) as 0.05. The design effect was taken as 2.

Data entry and analysis

Using SPSS Statistics, version 26.0 (IBM Corp., Armonk, NY), data were entered and analyzed. Qualitative factors were transformed into percentages and proportions. Data's normality was determined using the Kolmogorov-Smirnov and Shapiro-Wilk tests. Using binominal and multinominal linear regression, the relationship between dependent (protection against radiation exposure) and independent variables (such as fluoroscope awareness) was determined. In addition, Version 3.5 of PROCESS macro was employed for mediation analysis. p-values less than or equal to 0.05 were deemed statistically significant.

## Results

A total of 505 individuals participated in our healthcare survey. Out of these 505 individuals, 374 participants (74.1%) were male and 131 (25.9%) were females. Among these, 8 (1.6%) were professors, 15 (3%) were associate professors, 15 (3%) were assistant professors, 42 (8.3%) were senior registrars, 305 (60.4%) were residents and 120 (23.8%) were operation theatre assistants. About 429 individuals (85%) from the sample data performed or assisted with surgeries for five days in a week, 70 (13.9%) performed surgeries for four days, and 6 (1.2%) for three days. All the professors usually performed surgeries for four days. Among the associate professors, 3 (20%) performed surgeries for three days whereas 12 (80%) performed surgeries for four days. Among the assistant professors, 3 (20%) performed surgeries for three days whereas 12 (80%) performed surgeries for four days. Among the senior registrars, 9 (21.4%) performed surgeries for four days whereas 33 (78.6%) performed them for five days. Among the residents, 29 (9.5%) performed surgeries for four days, whereas 276 (90.5%) performed them for five days. All the OTAs assisted with the surgeries for five days; 2 (0.4%) individuals performed 5 surgeries in a week, 23 (4.6%) did 6-10 surgeries per week, 43 (8.5%) performed 11-15 surgeries per week while the remaining 437 (86.5%) individuals performed more than 15 surgeries per week. From the sample population, 284 (56.2%) were aware of the usage of the fluoroscope and 203 participants (40.2%) had read a research on fluoroscopy. About 225 individuals (44.6%) protected themselves from radiation during fluoroscopy by using a lead apron; 264 participants (52.3) used more than one one lead apron for protection whereas 16 participants (3.2%) used nothing.

**Figure 1:**
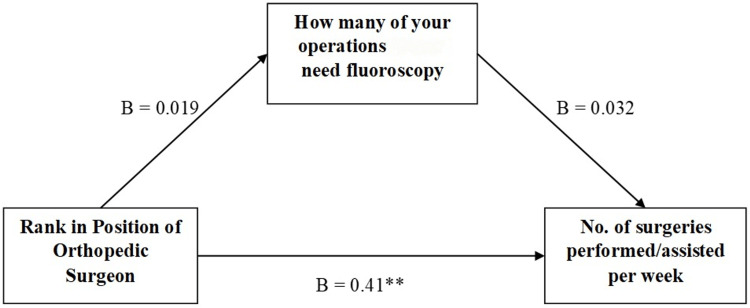
Effect of rank and the mediation effect of operations needing fluoroscopy on surgeries performed per week B indicates the regression coefficient. "No. of surgeries performed per week" is a dependent variable, "rank in position of orthopedic surgeon" is independent whereas "how many of your operations need fluoroscopy" is taken as the mediator. **p<0.001

Figure 1 indicates mediation model 4; the direct effect of the rank of orthopedic surgeons on the number of surgeries performed daily was significant. However, when using fluoroscopy usage in an operation theater as a mediator, the indirect effect of orthopedic surgeons on several surgeries performed daily was found to be insignificant.

**Figure 2:**
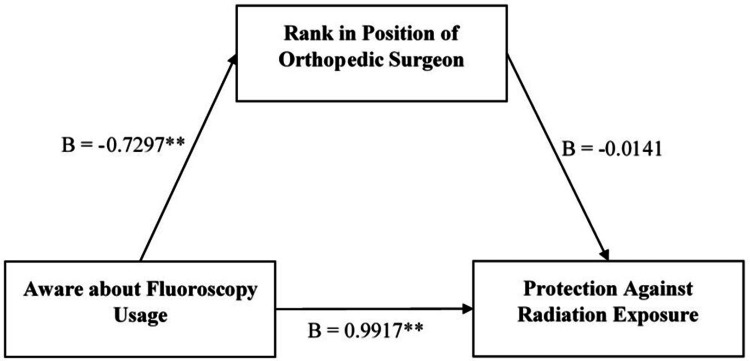
Effect of awareness and mediating effect of position of orthopedic surgeons on protection against radiation exposure B indicates the regression coefficient. "Awareness about fluoroscopy usage" is taken as an independent variable whereas "protection against radiation exposure" is taken as a dependent variable. "Rank in position of orthopedic surgeon" is taken as the mediator. **p<0.001

Awareness regarding fluoroscopy usage was significantly associated with protection against radiation exposure (B = 0.9917, CI: 0.9428, 1.0406), as shown in Figure 2. By taking the rank of an orthopedic surgeon as a mediating variable, an insignificant indirect relation (B = 0.0103) was found between the independent and dependent variables showing that position did not affect the relationship between the two variables.

**Figure 3:**
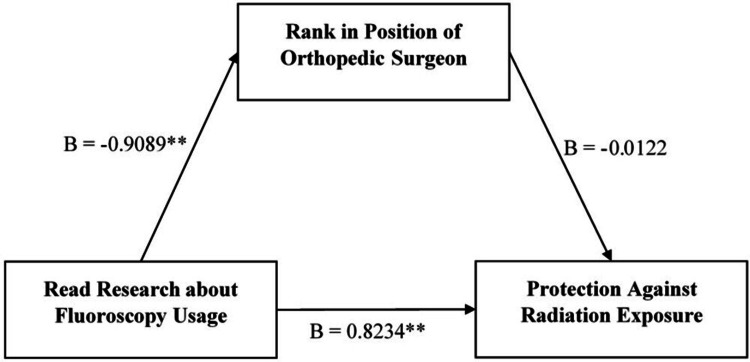
Effect of reading research and mediating effect of position of orthopedic surgeons on protection against radiation exposure B indicates the regression coefficient. "Read research about fluoroscopy usage" is taken as an independent variable whereas "protection against radiation exposure" is taken as a dependent variable. Position of orthopedic surgeons is taken as the mediator. **p<0.001

Reading research about fluoroscopy usage was significantly associated with protection against radiation exposure (B = 0.8234, CI: 0.7467, 0.9001), as shown in Figure 3. An insignificant indirect association was found between the two variables when taking the rank of orthopedic surgeons as a mediator (B = 0.0111).

## Discussion

Fluoroscopic imaging is vital in orthopedic surgery; however, it carries the danger of ionizing radiation exposure [11]. To limit this danger to the operator and colleagues in the operating room, adequate knowledge and awareness of radiation safety are required. This research indicated, however, that the degree of radiation safety education among orthopedic surgery personnel is poor.

Wilhelm Roentgen discovered X-rays in 1895, and since then, scientists have worked to improve the speed and quality of X-ray picture production. This resulted in the creation of the fluoroscopy apparatus [12]. The benefit of fluoroscopy over conventional X-rays is that it offers quicker results and displays the picture in real time throughout the process [13]. However, a significant disadvantage is that it exposes both the patient and surgeon to greater radiation levels than conventional X-rays that can lead to cataract formation, radiation dermatitis, skin cancer, and thyroid cancer. Women are also susceptible to acquiring breast cancer and exposing their unborn children to radiation [14]. Despite this, fluoroscopy has been a crucial tool in the operating room for orthopedic surgeons for many years for procedures such as the insertion of intramedullary nails for long bone fractures, reduction of closed fractures, removal of foreign bodies, external fixation of fractures, and insertion of hardware percutaneously [13].

There is a paucity of understanding among orthopedic residents, consultants, paramedics, and OT personnel about ionizing radiation and the associated equipment, according to research [15]. Only half of the participants in this survey were aware of the fluoroscope's use, and only 40.2% had read any literature on it. These numbers are very low, and this issue requires significant attention. These results are close to those of earlier researches that also showed that orthopedic residents, consultants, paramedics, and OT staff had a limited understanding of fluoroscopy and its radiation dangers [7,9]. According to the study done by Saroki et al., 91.2% of surgeons agreed that the majority of orthopedic surgeons need to be better educated about radiation safety [7].

In this research, 44.6% of participants used one apron to shield themselves from radiation during fluoroscopy, whereas 52.3% used several aprons. This takes the overall percentage to 96.9%, which is consistent with findings from a previous research. An academic unit performed an audit by mailing a questionnaire to 28 orthopedic physicians. The findings revealed that 96.4% of participants reported always wearing a lead apron [14]. A total of 98.4% of registrars reported using a lead apron in a separate survey [16]. This finding is comparable to that of a 2011 research published in the *Journal of Paramedical Sciences* conducted among radiographers at several hospitals in Hamadan City, Iran, where 98% of staff were aware of personal protection gear such as lead aprons and thyroid shields [17]. Only 78.9% of participants wore a lead apron, according to another research from Kerman, Iran [18]. Despite the increased danger of radiation exposure associated with wearing a broken lead apron, over 78% of registrars reported using one and said that it was the only option available.

During surgery, orthopedic surgeons are mostly exposed to scattered radiation, as opposed to direct radiation. The use of a lead apron and protective equipment for the testicles and thyroid gland may reduce the negative effects of radiation. Studies indicate that adequate protection may minimize a physician's exposure to radiation by up to 90% [19]. Compliance with body shields is often high, but other preventive measures, such as thyroid shields, are employed less frequently [20]. This research did not disclose data on the use of thyroid protectors in our environment, which represents a possible drawback. In addition, the use of dosimeters is not required as part of our radiation protection policy.

Since many radiation protective technologies are unavailable, it begs the issue of whether orthopedic doctors would have used them if they had been widely accessible. Moreover, in a research by van Papendorp et al., some participants were unaware that some equipment might be used for radiation protection, supporting the participants' lack of knowledge and awareness of radiation [15]. Meisinger et al. performed research that indicated various potential reasons why operators may not use particular radiation protective equipment. These included the uncomfortable placement of shields, weight of clothes, the tight fit of thyroid collars, and the rigidity of lead gloves. These results imply that the gadgets may be impractical and painful to use, necessitating additional development [20].

As this was a cross-sectional research study, no information was gathered on any awareness initiatives or their results and impacts. Also, no information was gathered about the potential causes for not using radiation protective gear. In addition, this survey was only done at tertiary care institutions, and thus, information about the knowledge of physicians in secondary care or primary care hospitals was not obtained. This investigation was done in just one Pakistani city. On a nationwide scale, we propose doing multi-center research. This will not only provide accurate statistics but also identify regions with the lowest awareness that need quick interventions.

In conclusion, we strongly recommend implementing the following actions to ensure radiation safety in the field: incorporating radiation safety knowledge into the training curriculum and examination, requiring registrars to use picture intensifiers under supervision, providing radiation protective clothing and equipment, and making the use of dosimeters mandatory. These measures are crucial for safeguarding the well-being of both healthcare professionals and patients, and their implementation will contribute to a safer and more effective radiology practice.

## Conclusions

The majority of orthopedic surgeons routinely employ fluoroscopic imaging during surgery; however, they typically lack a comprehensive grasp and awareness of the radiation safety risks connected with this technique. Personal safety equipment is often unavailable or underutilized when it is. Therefore, it is suggested that a radiation safety and protection training program be created within the orthopedic department. This program should cover the radiation dose limits recommended by the International Commission on Radiological Protection (ICRP). It should also teach the use of radiation protection devices and tools, like lead aprons and protective eyewear. Additionally, it should provide information on the correct techniques for using fluoroscopic imaging. It is recommended that a weekly class be held in the orthopedic department on the topic of fluoroscopy. Additionally, experts from the radiology department should be invited to conduct workshops on reducing radiation exposure.
